# Oxford Nanopore sequencing: new opportunities for plant genomics?

**DOI:** 10.1093/jxb/eraa263

**Published:** 2020-05-27

**Authors:** Kathryn Dumschott, Maximilian H-W Schmidt, Harmeet Singh Chawla, Rod Snowdon, Björn Usadel

**Affiliations:** 1 Institute for Biology I, BioSC, RWTH Aachen University, Aachen, Germany; 2 IBG-4 Bioinformatics, CEPLAS, Forschungszentrum Jülich, Jülich, Germany; 3 Department of Plant Breeding, Justus Liebig University Giessen, Giessen, Germany; 4 Institute for Biological Data Science, Heinrich Heine University Düsseldorf, Düsseldorf, Germany; 5 University of Essex, UK

**Keywords:** Basecalling, *de novo* assembly, gene annotation, MinION flow cell, Oxford Nanopore, third-generation sequencing

## Abstract

DNA sequencing was dominated by Sanger’s chain termination method until the mid-2000s, when it was progressively supplanted by new sequencing technologies that can generate much larger quantities of data in a shorter time. At the forefront of these developments, long-read sequencing technologies (third-generation sequencing) can produce reads that are several kilobases in length. This greatly improves the accuracy of genome assemblies by spanning the highly repetitive segments that cause difficulty for second-generation short-read technologies. Third-generation sequencing is especially appealing for plant genomes, which can be extremely large with long stretches of highly repetitive DNA. Until recently, the low basecalling accuracy of third-generation technologies meant that accurate genome assembly required expensive, high-coverage sequencing followed by computational analysis to correct for errors. However, today’s long-read technologies are more accurate and less expensive, making them the method of choice for the assembly of complex genomes. Oxford Nanopore Technologies (ONT), a third-generation platform for the sequencing of native DNA strands, is particularly suitable for the generation of high-quality assemblies of highly repetitive plant genomes. Here we discuss the benefits of ONT, especially for the plant science community, and describe the issues that remain to be addressed when using ONT for plant genome sequencing.

## Teaser

The sequencing of plant genomes is challenging due to their large size and abundant repetitive DNA, but third-generation platforms such as Oxford Nanopore Technologies can now generate high-quality assemblies.

## Introduction

DNA sequencing technology was introduced more than four decades ago and has evolved over time to produce data at ever-increasing rates. First-generation sequencing was established in 1977 when Sanger and Coulson published the first virus genome sequence, bacteriophage ϕX174 (Sanger *et al.*, 1977). First-generation sequencing dominated the field until the mid-2000s when high-throughput sequencing technologies, dubbed second-generation sequencing, emerged. The maximum read length of second-generation sequencing methods was typically shorter than for Sanger sequencing, but the higher throughput and relatively low cost made them competitive choices for large-scale sequencing projects (Lu *et al.*, 2016; Bolger *et al.*, 2019). These second-generation sequencing technologies remain popular for the analysis of simple genomes, resequencing, and RNA sequencing (RNA-seq), but the short reads they generate often lead to suboptimal assemblies, especially for *de novo* assemblies of large, highly repetitive genomes (Lu *et al.*, 2016).

The most recent developments in sequencing technology make it possible to obtain significantly longer reads while still generating data at faster rates than first-generation methods. These third-generation technologies sequence single DNA molecules in real time, and the reads can be many kilobases in length. Such reads can span the large repetitive regions of complex genomes, thus improving sequence assemblies (Lu *et al.*, 2016). Third-generation sequencing was spearheaded by Pacific Biosciences (PacBio) with their single-molecule real-time (SMRT) technology and was soon applied to plant genomes (VanBuren *et al.*, 2015). This was followed by the launch of Oxford Nanopore Technologies (ONT) in 2014 (Box 1). Here we discuss the current advantages and challenges of the third-generation ONT sequencing platform and its potential as a method of choice for the plant genome sequencing community.

**Box 1. Key developments in Oxford Nanopore Technologies application for plants**

**• One MinION flow cell can generate enough data to assemble a small plant genome**

Michael *et al.* (2018) report the assembly of a highly contiguous Arabidopsis genome using only one MinION flow cell. This study demonstrated that ONT technology can be used to assemble small plant genomes (i.e. <200 Mb) to an early draft stage using a single flow cell and with minimal effort.

**• Medium size plant genome assemblies are possible and competitive using ONT technology**

Schmidt *et al.* (2017) used ~135 Gb of ONT long-read data generated from 31 flow cells to assemble the genome of a wild tomato species to a high contiguity. This assembled genome was then compared with a related accession that had been sequenced and assembled using short reads. Given the higher output that can be obtained per flow cell and better read lengths using improved protocols, even quicker turnarounds may be possible today.

**• Medium to small plant genomes can be assembled and brought to chromosome scale using additional techniques**

Belser *et al.* (2018) showed that ONT data can be used to assemble a genome that can then be subsequently brought to chromosome scale using their case optical mapping. It can be expected that simpler techniques such as Hi-C (Feng *et al.*, 2014) would produce similar results.

**• Long reads generated from ONT flow cells are found to be useful for validating heterozygous genome assemblies**

Wang *et al.* (2020) sequenced and assembled a highly heterozygous eucalyptus genome using a combination of long read data generated from ONT and short read Illumina data. They demonstrate how ONT long read sequencing provides important information for *de novo* assemblies and use a 10% hold out strategy to assess different assembly pipelines that incorporate long read data.

## The potential of Oxford Nanopore Technologies sequencing for plant genomics

The release of the MinION platform in 2014 established ONT at the forefront of low-cost third-generation sequencing platforms. The MinION features a flow cell containing 2048 pores divided into four groups of 512, which are monitored by ONT software (Jain *et al.*, 2016). The MinION was quickly followed by the GridION (designed to run five MinION flowcells) and PromethION (designed to run 24 or 48 larger capacity flow cells), which utilize the same core technology as the MinION but are designed for larger sequencing loads.

Unlike PacBio, which is a ‘sequencing by synthesis’ platform, ONT uses a novel approach where native DNA molecules are pulled through nanoscale pores (nanopores) that accept only one DNA molecule at a time. As the DNA moves through the pore, sensors detect changes in the ionic current corresponding to the characteristics of each passing nucleotide. This information can be visualized in a ‘squiggle plot’ and provides the signal used for basecalling (Deamer *et al.*, 2016). Theoretically, sequencing continues until the end of the DNA fragment or until the pore becomes physically blocked, allowing for unprecedented read lengths that have the potential to significantly improve *de novo* genome assemblies and the detection of structural variations in large genomes. This is especially important in plant genomes, which contain highly repetitive regions derived from transposons and tandem repeats (Bolger *et al.*, 2019).

ONT has been used to sequence small genomes such as that of the bacterium *Escherichia coli* (Loman *et al.*, 2015), as well as large and repetitive plant and animal genomes. Examples include the human genome (Jain *et al.*, 2018) and plant genomes, ranging from the ~119.5 Mbp genome of *Arabidopsis thaliana* (Michael *et al.*, 2018) to the 2.53 Gbp genome of *Chrysanthemum nankingense* (Song *et al.*, 2018) (Table 1). ONT has also been used to improve the accuracy of single nucleotide polymorphism (SNP) genotyping in complex polyploid plant genomes, where low-coverage long-read sequencing achieves superior genome alignments (Malmberg *et al.*, 2019).

**Table 1.** Plant species sequenced using the ONT platform

Plant species	Genome size/N50	Sequencing technology	Assembler	Reference
*Arabidopsis thaliana*	119.5 Mbp/N50 12.3 Mbp (contig)	Illumina, ONT	Canu, Miniasm, Pilon	Michael *et al.* (2018)
*Anthoceros agrestis* (field hornwort)	116.9 Mbp/N50 155.5 kbp (contig) 17.3 Mbp (scaffold) (Bonn strain); 122.9 Mbp/N50 1.8 Mbp (contig) (Oxford strain)	ONT, Hi-C, Illumina (Bonn strain); ONT, Illumina (Oxford strain)	MaSuRCA, Pilon, HiRise (Bonn strain); Miniasm, Racon, Pilon (Oxford strain)	F.W. Li *et al.* (2020)
*Anthoceros punctatus*	132.8 Mbp/N50 1.7 Mb (contig)	ONT, Illumina	Miniasm, Racon, Pilon	
*Spirodela polyrhiza* (common duckweed)	138.49 Mbp/N50 3.34 Mbp (contig), 7.68 (scaffold)	ONT, Hi-C	Miniasm; Proximo (for Hi-C data)	Harkness *et al.* (2020, Preprint)
	139.7 Mbp/N50 2.9 Mbp (contig)	Illumina, ONT	Miniasm, Racon, Pilon	Hoang *et al.* (2018)
*Tectona grandis* (teak)	317 Mbp/N50 357 kbp (scaffold), 277 kbp (contig)	Illumina, Illumina Mate Pairs, ONT	MaSuRCA, SSPACE, GapCloser,	Yasodha *et al.* (2018
*Oryza sativa* L. (rice) IR64	367 Mbp/N50 1.6 Mbp (scaffold)	ONT, 10× Genomics	Supernova, Canu	Tanaka *et al.* (2020)
*Corylus avellana* L. (European hazel)	370 Mbp/N50 36.65 Mbp (scaffold)	Illumina, ONT, Hi-C	MaSuRCA, HiRise	Lucas *et al.* (2019, Preprint)
*Oryza sativa* (rice) Carolina Gold Select	377 Mbp/N50 1.72 Mbp (scaffold), N50 1.63 Mbp (contig)	ONT, Illumina	MaSuRCA, Flye	Read *et al.* (2020)
*Oryza sativa* (rice)	386.5 Mbp N50 6.32 Mbp (contig) (Basmati 334); 383.6 Mbp/N50 10.53 Mbp (contig) (Dom Sufid)	ONT, Illumina	Canu, Fly, Medaka, Pilon	Choi *et al.* (2020)
*Lupinus albus* (white lupin)	451 Mbp/N50 9.88 Mbp (scaffold), 7.11 Mbp (contig)	ONT, PacBio, Illumina, Bionano optical mapping	Canu, Falcon (for PacBio data only), Pilon, Bionano Solve	Hufnagel *et al.* (2019)
*Dioscorea dumetorum* (yam)	485 Mbp/N50 3.2 Mbp (contig)	ONT, Illumina	Canu, Racon, Pilon	Siadjeu *et al.* (2020)
*Juglans sigillata* (iron walnut)	536.5 Mbp/N50 16.43 Mbp (scaffold), N50 4.34 Mbp (contig)	ONT, Illumina, Bionano, Hi-C	Canu, wtdbg, Pilon	Ning *et al.* (2020)
*Juglans regia* (walnut)	547 Mbp/N50 31.49 Mbp (scaffold), 1.36 Mbp (contig)	ONT, Illumina short read, Hi-C	MaSuRCA, HiRise	Marrano *et al.* (2019 Preprint)
*Eucalyptus pauciflora* (snow gum)	594.87 Mbp/N50 3.23 Mb	ONT, Illumina	MaSuRCA	Wang *et al.* (2020)
*Brassica oleracea*	630 Mbp N50 29.5 Mbp (scaffold), 7.3 Mbp (contig)	Illumina, ONT, Bionano	Ra, (SMARTdenovo, wtdbg), Racon, Pilon, Bionano Solve and Access	Belser *et al.* (2018)
*Brassica rapa*	529 Mbp/N50 15.4 Mbp (scaffold), 3.8 Mbp (contig)			
*Musa schizocarpa*	587 Mbp/N50 36.8 Mbp (scaffold), 4.0 Mbp (contig)			
*Oryza coarctata* (wild rice)	665 Mbp/N50 1.86 Mbp (scaffold), 15.13 kbp (contig)	Illumina, ONT, Illumina Mate-Pair	PLATANUS, SSPACE, GapCloser	Mondal *et al.* (2018)
*Asparagus setaceus* (asparagus fern)	710.15 Mbp/N50 2.19 Mbp (scaffold)	ONT, Illumina, 10× Genomics, Hi-C	Canu, Pilon; LACHESIS (for Hi-C data)	S.F. Li *et al.* (2020)
*Euryale ferox* (prickly waterlily)	725.2 Mbp/N50 4.75 Mbp (contig)	ONT, Illumina, Hi-C	Canu, Pilon; LACHESIS (for Hi-C data)	Yang *et al.* (2020)
*Ceratophyllum demersum* (rigid hornwort)	733.3 Mbp/N50 1.56 Mbp (contig)			
*Sorghum bicolor* (sorghum)	732 Mbp/N50 33.28 Mbp (scaffold), 3.05 Mbp (contigs)	Illumina, ONT, Bionano	Canu, SMARTdenovo, Pilon, Nanopolish, Bionano	Deschamps *et al.* (2018)
*Cannabis sativa* (cannabis)	748 Mbp (1.39 Gbp F_1_ hybrid)/N50 742 kbp (contig) (172 kbp for F_1_ hybrid)	Illumina, PacBio, ONT	Miniasm, Racon, Pilon	Grassa *et al.* (2018, Preprint)
*Eriobotrya japonica* (loquat)	760.1 Mbp/N50 39.7 (scaffold)	ONT, Illumina, Hi-C	Canu, SMARTdenovo, Racon, Pilon; BWA and LACHESIS (for Hi-C data)	Jiang *et al.* (2020)
*Lonicera japonica* (Japanese honeysuckle)	843.2 Mbp N50 84.4 Mbp (scaffold)	ONT, Illumina, Hi-C	Canu, SMARTdenovo, Pilon; LACHESIS, SLR, SALSA (for Hi-C data)	Pu *et al.* (2020)
*Solanum pennellii* (wild tomato)	1.0 Gbp/N50 2.45 Mbp (contig)	Illumina, ONT	Canu, SMARTdenovo, Pilon	Schmidt *et al.* (2017)
*Chrysanthemum nankingense* (chrysanthemum)	2.53 Gbp/N50 130.7 kbp (contig)	Illumina, ONT	Canu, SMARTdenovo, Pilon	Song *et al.* (2018)

Additional benefits of the MinION include its low investment cost and portability. Currently, an ONT MinION starter pack is available for US$1000 (https://nanoporetech.com/products/minion). The MinION plugs into a normal laptop via USB 3.0 and the entire system weighs only 103 g, making it possible to sequence at any location with access to power and an internet connection. Sequencing has been carried out on the International Space Station (Castro-Wallace *et al.*, 2017), in the field to identify closely related plants in Snowdonia National Park (Parker *et al.*, 2017), on site in West Africa to analyse Ebolavirus samples (Quick *et al.*, 2016), and on farms in East Africa to identify strains of Cassava virus (Boykin *et al.*, 2018).

Even the larger ONT systems such as the GridION X5 and PromethION 24 (rental costs of US$49 995 and US$165 000, respectively) are significantly less expensive than competing platforms. For small-scale projects, costs can be further reduced by multiplexing samples on one MinION flow cell using a barcoding kit, or by using a Flongle adaptor that plugs into a MinION or GridION system, allowing for sequencing on even smaller flow cells. These contain 126 channels (compared with MinION’s 512) that can produce up to 2 Gb output in a run. The significantly lower start-up costs of ONT compared with its competitors mean that even smaller laboratories have the opportunity to generate their own third-generation sequencing data (Maestri *et al.*, 2019).

**Fig. 1.**
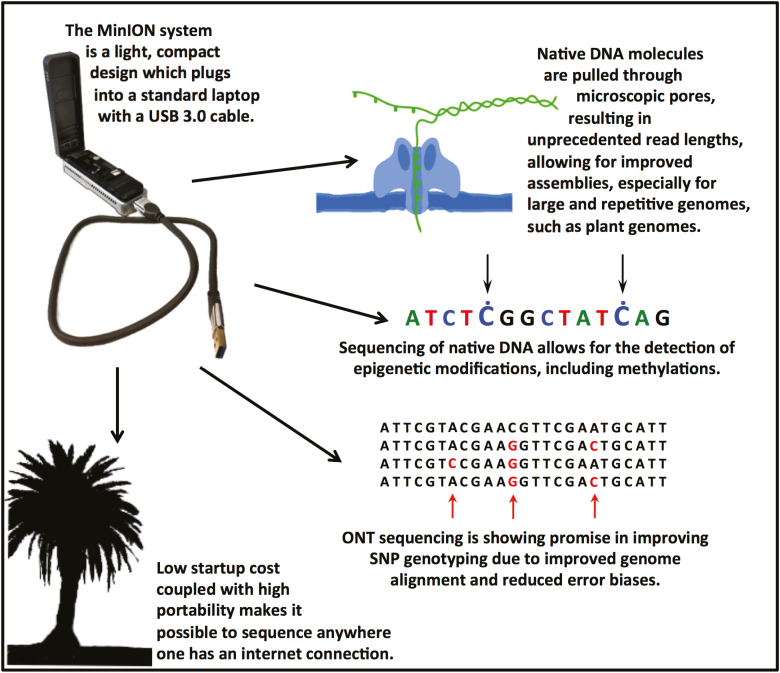
ONT offers a variety of important advantages to the wider plant genomics community.

One unique advantage of ONT is the ability to detect epigenetic modifications in native DNA (Jain *et al.*, 2016). DNA methylation detection (Rand *et al.*, 2017; Simpson *et al.*, 2017) was originally limited to methylated CpG dinucleotides (Shim *et al.*, 2013), but the technology has improved to include other DNA methylation states such as isolated 5mC and 6mA (Ni *et al.*, 2019). Additionally, Parker *et al.* (2019, Preprint) demonstrated that ONT can detect *N*^6^-methyladenosine in native *A. thaliana* RNA. ONT’s basecaller Guppy (from v3.2.1 onward) also allows certain DNA methylation sites to be called, such as 5mA, and 6mC in a CpG context, although it has currently only been trained on human and microbial data. A basecalling augmentation tool by ONT called Megalodon (https://github.com/nanoporetech/megalodon) can be combined with Taiyaki to train machine-learning algorithms (neural networks) for detecting plant-specific modifications. However, this requires additional data and significant computational resources such as graphics processing units (GPUs). Since DNA methylation plays a key role in the regulation of gene expression and in other cellular processes such as responses to stimuli (Law and Jacobsen, 2010), detecting these modifications during DNA sequencing provides valuable additional data (Simpson *et al.*, 2017). The investigation of CHG and CHH context-dependent methylation (Law and Jacobson, 2010) remains important, especially in plants. Whole-genome bisulfide sequencing is a widely adopted method for investigating these methylations. However, different approaches, which range from the experimental conditions to the downstream bioinformatics pipelines, make it difficult to compare studies between research groups (Zhang *et al.*, 2018), highlighting the potential advantages of ONT as a standardized method for detecting native DNA methylation (Fig. 1).

## The challenges of Oxford Nanopore Technologies sequencing for plant genomics

**Fig. 2.**
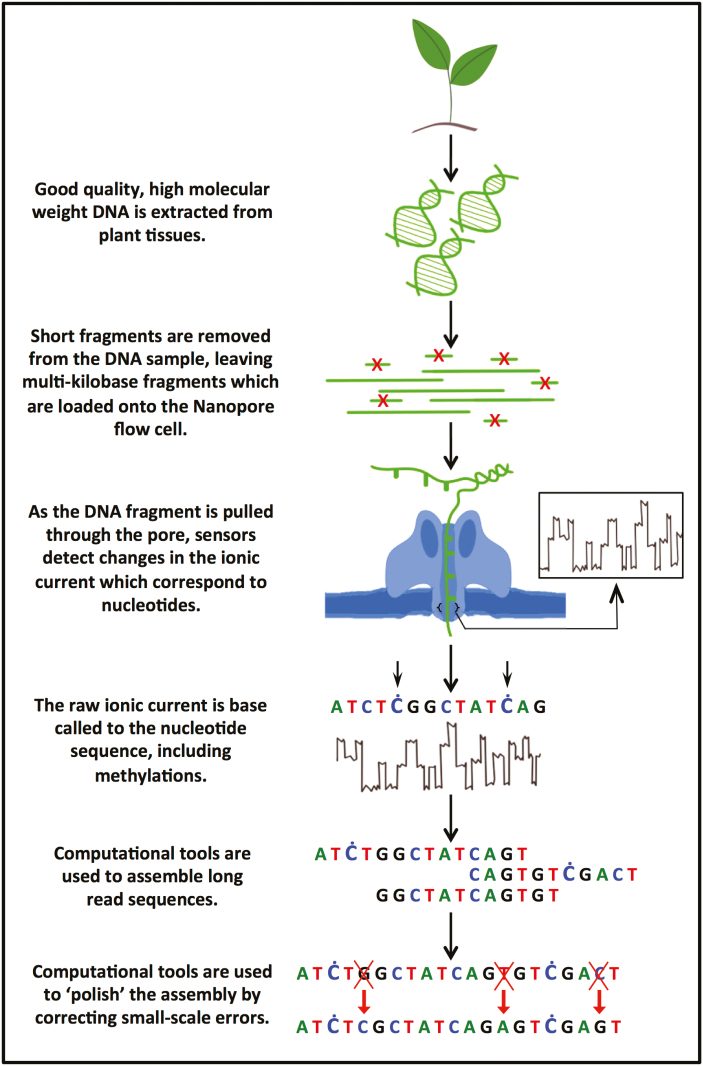
From plant tissue to genome assembly: the main steps in ONT sequencing. Optimizing each step can significantly increase the sequencing output and assembly quality.

Although ONT is already established at the forefront of third-generation sequencing, several limitations of the technology remain, especially for sequencing highly repetitive plant genomes (Jiao and Schneeerger, 2017). Large amounts of high-quality DNA are required for a successful ONT sequencing run, defined as a high yield run with long reads (Schmidt *et al.*, 2017). However, extracting intact high molecular weight DNA from plants is hindered by cell walls and secondary metabolites, with residual metabolites also remaining bound to the DNA, reducing sequencing yields (Schalamun *et al.*, 2019; Vaillancourt and Buell, 2019, Preprint). There is often an inverse correlation between the quality and quantity of extracted DNA (Schalamun *et al.*, 2019), and multiple DNA extraction protocols should be tested and optimized before sequencing a new plant species (Fig. 2; Table 2).

**Table 2.** Current challenges and solutions when using ONT to sequence plant genomes

Challenge	Potential solutions
Low DNA quality and quantity	Test multiple extraction protocols and optimize for each plant species.
Short read contamination	Removal of short and medium-sized fragments using BluePippin Prep or Circulomics Short Read Eliminator kits, the latter being easier to use.
Basecalling speed and computational requirements	PromethION includes the hardware needed for fast basecalling. MinION basecalling time can be significantly reduced by using GPUs.
Long assembly computation time	Newer assemblers can significantly reduce computational time (e.g. wtdbg2).
Remaining uncorrectable base errors	Additional Illumina sequencing and polishing is currently required (Watson and Warr, 2019). This might be addressed with newer pore versions or basecalling models trained for particular species. Useful software includes Racon and Pilon.
Assembly is not (near) chromosome scale	Additional techniques such as optical mapping or Hi-C can be used to order and place contigs and obtain (near) chromosome-scale assemblies, at least for small and medium-sized plant genomes.
Genome structural and functional annotation	For structural annotation, long-read technology can be used with programs such as Stringtie2 (Kovaka *et al.*, 2019). For functional annotation, free online tools relying on specific plant expertise are available, such as Mercator (Schwacke *et al.*, 2019), TRAPID (Van Bel *et al.*, 2013), or Hayai (Ghelfi *et al.*, 2019), in addition to general tools such as Blast2GO (Götz *et al.*, 2008). The plant repeat database (Nussbaumer *et al.*, 2013) can be used to analyse repetitive DNA, and structural variations can be analysed using NGMLR/sniffles (Sedlazeck *et al.*, 2018).

**Fig. 3.**
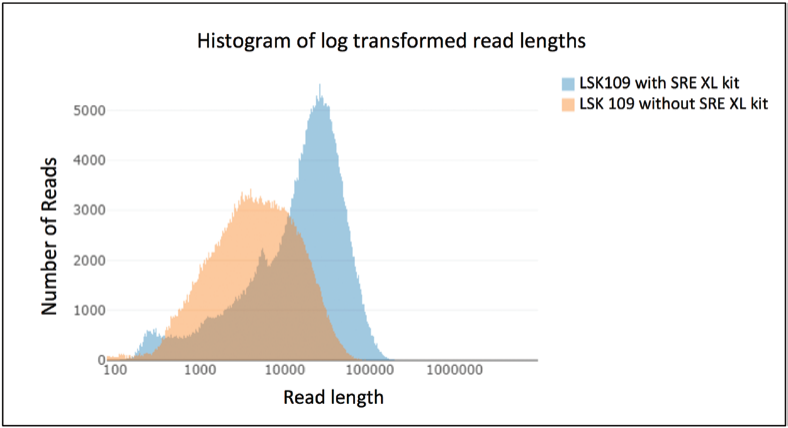
Difference in read lengths between an untreated sample and a sample treated with the Circulomics Short Read Eliminator kit. DNA was extracted from rapeseed (*Brassica napus*) and sequenced on an ONT MinION (image created using NanoComp by De Coster *et al.* (2018).

It is important to generate read lengths that span complex, repetitive DNA segments. Various protocols can be used to remove short DNA fragments, the easiest of which involves an adjustment to the quantity of NaCl and polyethylene glycol (PEG) used during bead clean-up steps (Schalamun and Schwessinger, 2017). An alternative is nuclear extraction followed by electrophoretic size selection, using equipment such as the Sage Science BluePippin Prep method (Schmidt *et al.*, 2017). Although BluePippin achieves a clean size cut-off, sample recovery can be <50%, meaning that large quantities of input DNA are required. Furthermore, this method involves a substantial capital investment and recurring costs for consumables. A newer method for depleting short fragments is the Short Read Eliminator kit from Circulomics. Adopting a similar approach to bead clean-up, this kit relies on the precipitation of large DNA fragments, which are pelleted by centrifugation, while the shorter fragments remain in solution and are discarded (Fig. 3).

The correction of random read errors in the PacBio system is achieved using the circular consensus read technology that re-reads circularized DNA molecules multiple times, which are combined to produce high-fidelity results (Vollger *et al.*, 2020). Because ONT reads are not circularized, an analogous read consensus option is not available beyond 1D^2^ sequencing, which aims to sequence both strands. Therefore, ONT sequences still have markedly higher error rates compared with second-generation sequencing platforms. This reflects the low signal-to-noise ratio of ONT sequencing, which remains a key challenge (Rang *et al.*, 2018). Several factors contribute to this, including structural similarities between nucleotides and multiple nucleotides concurrently influencing the signal (Rang *et al.*, 2018). ONT therefore developed the flip–flop basecalling model, which uses two overlapping windows to interpret the raw signal. Nucleotides containing methyl groups or other modifications will also modify the signal, making basecalling more difficult.

An additional factor that significantly influences signal quality is the speed at which the DNA strand moves through the pore, as signal strength depends on the time each nucleotide resides within the sensing region. ONT chemistry therefore includes the attachment of a motor protein to the DNA, which slows the translocation of the nucleotides through the pore signalling region, improving signal quality and robustness (Rang *et al.*, 2018). However, the translocation speed of the motor protein can be sequence dependent, generating inconsistent signals especially in atypical segments such as homopolymer runs and multiple short repeats.

A comprehensive study on the basecalling accuracy of different sequencing platforms was performed using sequencing data from the bacterium *Klebsiella pneumoniae* (Wick *et al.*, 2019). Even with the best standard basecallers, read identity was just below 90%, whereas consensus accuracy was 99.4%. This can make the assembly of plant genomes more difficult than animal genomes, because the former tend to contain more repetitive DNA and are more likely to be polyploid (Jiao and Schneeberger, 2017). In part, this reflects the fact that ONT’s basecaller Guppy is only trained on PCR, human and bacterial data, resulting in a lack of optimization for native plant DNA containing side chain modifications. This contributes to the significantly lower quality scores of plant ONT data compared with data from other domains, and hinders downstream alignment and assembly pipelines.

As discussed above, an alternative approach that could address this challenge is the development of plant-specific basecalling models generated using the ONT tool Taiyaki. Wick *et al.* (2019) achieved consensus accuracy >99.9% with *K. pneumoniae* after training Taiyaki using *Klebsiella*-specific models. A major improvement was that the self-trained models accounted for base read errors caused by DNA methylation. From a hardware perspective, the new R10 pore, which facilitates a longer read-head design, promises higher raw read accuracy. Improvements to the accuracy of ONT basecallers rely solely on software improvement and can be applied retrospectively to existing ONT sequencing data.

## From Oxford Nanopore Technologies reads to genomes and useful data

As ONT sequencing technology continues to improve, the computational tools used to analyse raw sequencing data must also be optimized (Rang *et al.*, 2018). One key post-sequencing step is the translation of the electrical current output signal into the nucleotide sequence, which is the technological principle of basecalling. The latest improvements in ONT basecallers require GPU computing for the rapid processing of raw data (Nobile *et al.*, 2017), significantly improving basecalling speed compared with CPU-based workstations (Wick *et al.*, 2019). Although such GPU resources are made available through national/international service providers such as iPLANT/CyVerse or ELXIR/de.NBI (Tauch and Al-Dilaimi, 2019), it may nevertheless be advisable to invest in standard NVIDIA graphics cards, which are known to support high basecalling speeds. Consequently, the PromethION comes with enterprise-grade GPU computing installed. For MinION and Flongle, ONT has developed the MinIT and Mk1C for data acquisition and basecalling, eliminating the need for any external hardware. The alternative basecaller Chiron, developed by Teng *et al.* (2018), achieves throughput of only a few thousand bases per second despite running on GPUs, making it too slow for typical plant sequencing projects.

### Assembly

Several toolkits and pipelines are available for genome assembly (Fig. 2). One example, Canu, is based on the overlap layout consensus (OLC) principle (Koren *et al.*, 2017). Canu uses a ‘correction then assembly’ strategy, making it also useful as a pre-processing tool before switching to another assembler. One consideration when assembling larger plant genomes is that Canu needs to run on computer clusters and still requires significant run time (Schmidt *et al.*, 2017).

Similarly, MECAT (Xiao *et al.*, 2017) first corrects reads and then uses the basic Canu engine for genome assembly, although Canu was replaced with a string graph assembler in the more recent version, MECAT2. A string graph assembler is also used in NECAT (Chen *et al.*, 2020, Preprint), which has been adopted by ONT. However, both MECAT2 and NECAT still require initial read error correction as part of their assembly pipeline. Alternative OLC assemblers such as Ra (Vaser and Šikić, 2019, Preprint) and Miniasm (Li, 2016) directly assemble raw, uncorrected reads.

A number of alternative long-read assemblers have also been successfully applied to plant genomes (Schmidt *et al.*, 2017; Belser *et al.*, 2018; Wang *et al.*, 2020). These include SMARTdenovo and its successor wtdbg2/Redbean (Ruan and Li, 2020), the latter using fuzzy de Bruijn graphs as a more error-tolerant extension of the de Bruijn graph data structure typically used to assemble Illumina sequencing data. Another example, Flye, relies on a repeat graph data structure that also tolerates more sequencing errors (Kolmogorov *et al.*, 2019). In addition to these long-read assemblers, hybrid assemblers that use short, low-error sequences coupled with more error-prone long-read data are also available. One example is MaSuRCA (Zimin *et al.*, 2013), which can be slow when applied to complex plant genomes but has nevertheless been tested successfully in plant species, including the annual grass *Aegilops tauschii* (Zimin *et al.*, 2017).

### Polishing and consensus

Although recent advances in assembly algorithms have improved consensus handling, it is often still necessary to post-process the assembly before biological analysis (Fig. 2). Typically, ONT reads are used to correct the assembly as an additional consensus step. This can be achieved rapidly using Racon, which realigns the reads and should therefore provide good consensus accuracy (Vaser *et al.*, 2017). Racon is currently undergoing modifications to increase its speed by making it GPU compatible. However, Nanopolish can usually achieve superior accuracy by utilizing the original signal level traces rather than basecalled reads (Loman *et al.*, 2015). Even so, at least in the case of bacteria (Wick *et al.*, 2019), a custom-trained basecaller provided such high consensus accuracy after Racon-based polishing (>99.9%) that additional Nanopolish processing actually reduced the accuracy. Machine learning can also be used to correct errors. The ONT program Medaka (https://nanoporetech.github.io/medaka/benchmarks.html#evaluation-across-samples-and-depths) promises to outperform Racon and Nanopolish in terms of speed and accuracy for bacterial sequences, although it is currently trained only on bacterial and human data. Alternatively, the community-developed tool HELEN uses a similar approach, but is currently only trained on human data (Shafin *et al.*, 2019, Preprint).

It is also necessary to correct assemblies using an orthogonal technology, such as Illumina sequencing, to remove remaining small-scale sequence errors. The Pilon polisher is often used for this purpose (Walker *et al.*, 2014), following autocorrection of the assembly using ONT reads. This is because the best consensus accuracy of ≥99.9% is still not sufficient to achieve the minimum 99.99% base accuracy benchmark defined for a ‘finished human genome assembly’ or the actual accuracy of ~99.999% achieved by the International Human Genome Sequencing Consortium (2004). This level of accuracy is necessary because errors can significantly affect downstream protein prediction and subsequent interpretations (Watson and Warr, 2019). However, the technology is developing rapidly and it may not be appropriate to test old results against such benchmarks (Koren *et al.*, 2019). Nevertheless, efficient error correction is important, and even high-quality reference genomes may lack genes due to assembly problems, regardless of which sequencing technology was used.

### Assembly pipeline, improvement, and quality control

Researchers have a variety of options for data processing and *de novo* genome assembly, and some combinations are better than others depending on parameters such as data volume, genome size, and the heterozygosity and ploidy of the plant species. One approach, used by Schmidt *et al.* (2017) and Belser *et al.* (2018), is to first correct reads using Canu (Koren *et al.*, 2017) followed by assembly using SMARTdenovo (J. Ruan, unpublished github) and polishing with Illumina data using Pilon (Walker *et al.*, 2014). If available computational resources are not sufficient for Canu, Deschamps *et al.* (2018) showed that, at least for medium-sized genomes, the Canu correction step can be omitted.

The resulting assemblies can be scaffolded to near chromosome scale using Bionano optical mapping technology (Belser *et al.*, 2018; Deschamps *et al.*, 2018). The latter also carried out post-scaffolding polishing with ONT data using Racon (Vaser *et al.*, 2017) and 10× genomics data using the Long Ranger ALIGN pipeline to resolve medium-sized structural errors that Pilon could not fix before scaffolding

The need for polishing and overall assembly quality can be assessed using BUSCO, a tool that provides quantitative measures for genome completeness based on the anticipated gene content (Waterhouse *et al.*, 2018). Unpolished long-read assemblies often contain large numbers of small indels; hence many genes are not detected during BUSCO analysis. Polishing with tools such as Racon, Nanopolish, or Pilon will resolve these indels and increase the completeness score in BUSCO. Another approach for quality assessment is the LTR Assembly Index (LAI), which checks for the presence and integrity of long terminal repeats (LTRs) in the genome assembly (Ou *et al.*, 2018). LAI is therefore complementary to BUSCO because it uses the non-genic parts of the assembly, further evaluating the quality of genomes (Ou *et al.*, 2018).

### Gene calling and other forms of downstream analysis

As the ONT platform and associated gene assembly tools continue to develop, there will be a shift towards the downstream analysis of gene platforms, especially for gene calling. Pipelines such as MAKER-P (Campbell *et al.*, 2014) and BRAKER2 (Hoff *et al.*, 2016) are already available, but require computational resources and effort in model training. However, given ongoing developments in ONT for RNA-seq analysis (both full-length cDNA and native RNA), and more widespread adoption of PacBio’s full-length self-corrected RNA-seq analysis (dubbed ‘isoseq’), we are likely to see a move towards evidence-only-based gene finders, such as Stringtie2 (Kovaka *et al.*, 2019), which rely on long-read RNA/cDNAs. One limitation of Stringtie2 is that only genes corresponding to RNAs expressed with high enough coverage are detected. Unlike gene finding, gene functional annotation has already made the switch to high-throughput automated analysis using tools such as Mercator, TRAPID, or Hayai (Van Bel *et al.*, 2013; Ghelfi *et al.*, 2019; Schwacke *et al.*, 2019) as well as generalists such as Blast2GO (Götz *et al.*, 2008) to allow for the coming wave of ultra-large genome projects encompassing thousands of species (Lewin *et al.*, 2018).

## Conclusions and future directions

Many plant genomes are large and complex with highly repetitive regions, making it difficult to generate high-quality assemblies using first-generation or even second-generation sequencing methods (Bolger *et al.*, 2014; Jiao and Schneeberger, 2017). The increasing quantity and quality of long-read sequence data from low-cost ONT platforms therefore provide confidence for the success of future plant genome sequencing projects, which will lead to significant advances in plant genome and pangenome assemblies. Current challenges in areas such as read error rates will be overcome by the rapid advances of third-generation technologies, and the advantages of ONT already outweigh the shortcomings. In the future, ONT is set to provide unprecedented insight into the complexities of plant genomes, while ongoing developments for modified basecalling will also provide a sound basis for epigenomic and transcriptomic analysis.
